# A Practical Treatise on Nervous Exhaustion

**Published:** 1880-03

**Authors:** 


A PRACTICAL TREATISE ON NERVOUS EXHAUSTION (NEURASTHENIA.)—Its symptoms, nature, sequences, treatment. By George M. Beard, A.M., M.D., Fellow of the New York Academy of Mediciue; of the New York Academy of Sciences etc., etc. New York. William Wood & Co., 27 Great Jones Street. 1880.

The author in 168 pages seeks to give the symptoms, nature and treatment of what, we must confess, seems to be an imaginary disease, without cause or palpable origin. It is true insufficient nutrition is given as the cause, but of course innutrition leads to failure in the performance of the function of all organs and requires attention to general nourishment instead of special neurotic treatment. Nervous prostration or neurasthenia, as the author terms it, would seem to be the result of something, and that something we should seek, and endeavor to remove it. He finally comes to the point on page 179, and we agree with him fully when he says, "In quite a number of cases of neurasthenia no satisfactory relief or cure can be obtained until we first relieve the local disease from which the general neurasthenia originated and by which it is maintained. The irritation of the digestive and reproductive apparatus is reflected to the brain, the spine, the eyes, the ears, and by them to the whole system." And again on page 180 he says, "Perseverance is an element which is required both on the part of the physician and the patient. Diseases of many years standing are not to be driven from their strongholds by a single prescription, or by a brief and temporary respite from care." These sentences form redeeming features in the book and lead to a conclusion which the preceding pages would not justify.

The work is well enough, taking the above, and the like as a summation of the whole matter. With the understanding that the symptoms of neurasthenia as given in the first chapters of the book are to be considered, not as a disease but as reflex impressions of local disease, we have no objections to offer.

